# Sequence Comparison for Non-Enhanced MRA of the Lower Extremity Arteries at 7 Tesla

**DOI:** 10.1371/journal.pone.0086274

**Published:** 2014-01-16

**Authors:** Sören Johst, Stephan Orzada, Anja Fischer, Lena C. Schäfer, Kai Nassenstein, Lale Umutlu, Thomas C. Lauenstein, Mark E. Ladd, Stefan Maderwald

**Affiliations:** 1 Erwin L. Hahn Institute for Magnetic Resonance Imaging, University Duisburg-Essen, Essen, Germany; 2 Department of Diagnostic and Interventional Radiology and Neuroradiology, University Hospital, University Duisburg-Essen, Essen, Germany; Northwestern University Feinberg School of Medicine, United States of America

## Abstract

In this study three sequences for non-contrast-enhanced MRA of the lower extremity arteries at 7T were compared. Cardiac triggering was used with the aim to reduce signal variations in the arteries. Two fast single-shot 2D sequences, a modified Ultrafast Spoiled Gradient Echo (UGRE) sequence and a variant of the Quiescent-Interval Single-Shot (QISS) sequence were triggered via phonocardiogram and compared in volunteer examinations to a non-triggered 2D gradient echo (GRE) sequence. For image acquisition, a 16-channel transmit/receive coil and a manually positionable AngioSURF table were used. To tackle B_1_ inhomogeneities at 7T, Time-Interleaved Acquisition of Modes (TIAMO) was integrated in GRE and UGRE. To compare the three sequences quantitatively, a vessel-to-background ratio (VBR) was measured in all volunteers and stations. In conclusion, cardiac triggering was able to suppress flow artifacts satisfactorily. The modified UGRE showed only moderate image artifacts. Averaged over all volunteers and stations, GRE reached a VBR of 4.18±0.05, UGRE 5.20±0.06, and QISS 2.72±0.03. Using cardiac triggering and TIAMO imaging technique was essential to perform non-enhanced MRA of the lower extremities vessels at 7T. The modified UGRE performed best, as observed artifacts were only moderate and the highest average VBR was reached.

## Introduction

For the diagnosis of diseases of the lower extremity vasculature, digital subtraction angiography (DSA) is increasingly performed only in cases where contrast-enhanced MR angiography (CE MRA) has showed positive findings [1]. Recent publications indicate, however, that especially patients with severe renal dysfunction should not be examined with Gadolinium (Gd)-based contrast agents due to reported cases of Nephrogenic Systemic Fibrosis (NSF) after the administration of such contrast agents [2], [3]. Accordingly, in recent years several publications have focused on the evaluation of non-enhanced MRA sequences as an alternative to DSA/CE MRA for the diagnosis of lower extremity vascular diseases [1], [4]–[8].

It has been shown that non-enhanced MRA techniques seem to profit from 7T, especially in the head [9], [10]. But in order to perform non-contrast-enhanced MRA at 7T, one has to counter challenging issues such as B_0_ and B_1_ inhomogeneities [11], [12], particularly when considering body imaging [13], [14]. To tackle the latter issue, a recently published technique, Time-Interleaved Acquisition of Modes (TIAMO) [15], [16], was used for this study. The principle is to excite at least two different B_1_ transmission modes using static radiofrequency (RF) shimming in an interleaved acquisition. Overall signal homogeneity can be improved by exploiting the complementary radiofrequency patterns of the different transmission modes. Data from e.g. two acquisitions are not just averaged, but reconstructed the same way as if twice the number of coils would have been used. By that considerably more homogeneous images can be created.

Further, for non-enhanced MRA at 7T there is a need for heartbeat triggering due to a periodic variation in the signal intensity of the arteries that was observed in [17]. The most likely reason is interference between image acquisition frequency and heartbeat frequency. Using cardiac triggering combined with single-shot sequences acquiring the center of k-space at approximately the same time point of the cardiac cycle should be possible and thereby reduce the observed signal variations. In clinical routine at lower field strengths, ECG triggering is commonly used. Unfortunately, ECG triggering is severely limited at 7T due to an elevated T wave and other interferences [18]. To avoid this issue, acoustic cardiac triggering can be used [19]–[21].

**Figure 1:**
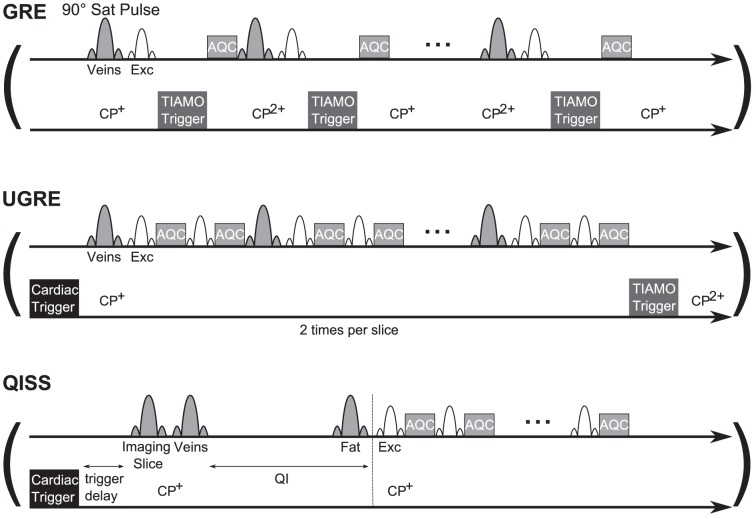
Sequence diagrams of GRE, UGRE and QISS. In GRE, no cardiac triggering is used. Here, the TIAMO trigger changes the excitation mode directly after every excitation pulse, which means that both modes needed for a complete slice are acquired in an interleaved fashion. Saturation pulses are applied every TR. In UGRE, the cardiac trigger event starts the acquisition of a complete slice in a single shot. Due to TIAMO, this acquisition has to be repeated to acquire the same slice with the second mode. Venous saturation RF pulses are applied only sparsely. QISS uses three different saturation pulses to prepare the image contrast: In the imaging slice to suppress background tissue, a travelling venous saturation pulse below the imaging slice and a fat saturation pulse in the imaging slice. After the venous saturation, a time interval QI allows unsaturated arterial blood spins to enter the imaging slice. No saturation pulses are applied during the single-shot slice acquisition. The duration of the trigger events is shorter than pictured in these diagrams.

In [17] a 2D GRE sequence capable of TIAMO imaging was used. Here, a trigger pulse changes the excitation mode directly after every excitation pulse which means that both modes needed for a complete slice are acquired in an interleaved fashion (Fig. 1 top). Venous saturation pulses were applied every TR. MRA image contrast is created by saturating the background tissue via the excitation RF pulses and by inflowing unsaturated arterial blood spins which provide hyperintense signal. In this study, to avoid the reported blood signal fluctuations, two single-shot sequences were chosen to be combined with cardiac triggering:

1. An Ultrafast Spoiled Gradient Echo (UGRE) sequence (a modified turboFLASH sequence without T1 contrast preparing inversion RF pulse) where the cardiac trigger starts the acquisition of a complete slice. This is repeated for the second TIAMO mode after the next cardiac trigger event. Venous saturation pulses are applied only sparsely (Fig. 1 middle). Image contrast is created the same way as in GRE, but UGRE is designed for quite fast 2D single-shot acquisitions.
2. A variant of the Quiescent-Interval Single-Shot (QISS) [22] sequence which is available as vendor-provided work in progress and used at 1.5T. Here, three 90° saturation RF pulses prepare the image contrast. The first one saturates the tissue signal in the imaging slice, the second one suppresses the veins and the third one suppresses fat signals. After the second preparation pulse, a time interval QI is introduced to give the unsaturated arterial blood spins time to enter the imaging slice before one complete slice is acquired (Fig. 1 bottom). As the QISS sequence is a vendor-provided work-in-progress at 7T, no source code was available and TIAMO could not be implemented. Both UGRE and QISS were triggered via phonocardiogram and compared in volunteer examinations to a non-triggered 2D gradient echo (GRE) sequence similar to the one used in [17].

The rationale of choosing UGRE and QISS is that both implementations used here are based on a GRE readout - techniques based on steady-state free precession (SSFP) like [6] are challenging at 7T due to the inherent B_0_ inhomogeneities). QISS produces promising results at lower field strength [5], but relies on multiple preparation pulses which might be less effective at 7T due to the inherent B_1_ inhomogeneities. In contrast to QISS, the implemented version of the UGRE uses just inherent saturation effects of the GRE readout and no preparation RF pulses to generate the basic MRA contrast. This might provide sufficient contrast even in areas were desired flip angles cannot be met. Additionally, UGRE is more time-efficient because no time interval QI is necessary. By that, higher flexibility in dealing with different heart rates should be possible.

## Materials and Methods

### Ethics Statement

The study was conducted in conformance with the Declaration of Helsinki and approved by the Ethics Commission of the Medical Faculty of the University Duisburg-Essen (study number 11-4898-BO). Written informed consent was obtained from each volunteer before the examination.

### Measurement configuration

Examinations were performed on a 7T whole-body system (Magnetom 7T, Siemens, Erlangen, Germany) with a 16-channel transmit/receive coil based on [23]. Five stripline meander elements were placed dorsally on the patient table and eleven meander elements were placed on a rigid, semicircular former above the table. A RF shimming system with 8 channels similar to [24] was used to drive the coil with an 8-channel variable power combiner (VPC) interfaced to a 16-channel Butler matrix. Eight of the 16 inputs of the 16-channel Butler matrix corresponding to the highest transmit signal in the corresponding transmit modes were connected to the VPC [23], [25]. In this way the 8-channel transmit system could be used to drive a 16-channel coil. The same configuration as in [17] was used.

Image acquisition was performed with a AngioSURF table [26], [27] with 2 m length by positioning the volunteers feet-first supine and manually moving the AngioSURF table to all required body positions through the RF coil, which remained stationary at the isocenter connected to the original patient table. To reduce total acquisition time (TA), a general B_0_ shim that was determined at the uppermost station was used for all stations [17].

For compliance with the International Electrotechnical Commission (IEC) guidelines, SAR calculations (CST Microwave Studio, Darmstadt, Germany) were performed in human adult male and female body models of the Virtual Family and the Visible Human [28], [29]. Full-wave simulations were applied with exact dimensions and characteristics of the 16-channel RF coil, and maximum permitted input power levels for each station were calculated from the simulations of the corresponding body models. Based on these simulations, a standardized SAR file was integrated into the SAR monitoring system [24].

Periodic vessel signal fall-offs over short segments, particularly observable in coronal MIP images, were reported in [17]. To counter this issue, triggering with a phonocardiogram [19]–[21] was used for UGRE and QISS imaging. As both sequences acquire individual slices in single-shot acquisition started by the cardiac trigger event, the center of k-space can be acquired in approximately the same time point of the cardiac cycle. To compare the impact on the artifact, GRE is acquired without cardiac triggering.

Consistent signal loss in the middle third of the thigh were observed in [17], which is suspected to be caused by RF wave interferences within the FOV due to the variable anatomy of pelvis and legs along the longitudinal axis. In those volunteers where the aforementioned problem was encountered, dedicated TIAMO shims [16] were calculated based on acquired B_1_^+^ maps in the same imaging position. The two shims were calculated to adjust the phases of the 8 transmit channels in such a way that in one shim the B_1_^+^ was maximized in a circular region of interest around the right superficial femoral artery, while in the other shim the B_1_^+^ in a circular region of interest around the left superficial femoral artery was maximized. The remaining stations were acquired by combining the CP^+^ and CP^2+^ modes, the first- and second-order circularly-polarized modes.

### Sequence modifications

TIAMO imaging was implemented in the UGRE sequence code by enabling trigger pulses that cause the RF shimming system to switch between the different modulator states. As the UGRE protocol is intended to acquire one slice in a single shot between two heartbeats, the TIAMO trigger was set after each acquired imaging slice. In this way, the same slice is acquired with the two imaging modes sequentially before the next slice is excited and acquired with both modes sequentially.

Additionally, the UGRE sequence had to be further modified. RF pulses for the suppression of the venous system were implemented into the sequence. To minimize the duration of the protocol and to be able to adjust the required TR to the heartbeat rate of the volunteers, saturation pulses could be applied sparsely (i.e. only every n-th line of k-space, where n can be flexibly chosen in the protocol). The normally used saturation pulse was exchanged with another sinc pulse with lower time-bandwidth product (TBWP) (3.6 instead of 7.8). Thus, a shorter pulse was achieved without increasing its SAR contribution at the cost of the exactness of the excitation profile, which is not relevant as long as the suppression ability is not affected. A flexible RF saturation pulse duration was implemented between 800 µs and 5000 µs with a default value of 3840 µs. For further reduction of the TR, the duration of the spoiling gradients following the saturation RF was made flexible (500 µs to 2000 µs).

As the QISS sequence is a vendor-provided work-in-progress at 7 T, no source code was available and no modifications could be implemented. Hence, TIAMO imaging could not be realized, and the protocol was run with the CP^+^ mode of the 16-channel coil and 1 average instead of the two or more that are necessary for TIAMO. The original QISS sequence proposed by Hodnett et al. [5] used a steady-state free precession (SSFP) readout. In this study, a GRE readout was used to avoid known artifacts and SAR limitations of SSFP at 7T [30].

### Imaging protocols

All protocols used a nominal flip angle of 80° and a FOV of 384 mm by 288 mm to acquire 60 transversal slices with 2 mm thickness using a matrix of 384 by 288 pixels and thus an in-plane resolution of 1 mm^2^. Parallel image acceleration with a GRAPPA factor of 4 utilizing 32 integrated auto-calibration lines was used. To account for inaccuracies in the manual positioning of the AngioSURF table, the different stations were scanned with an overlap that for an exact shift would lead to the lowermost 10 slices being overlapped with the uppermost 10 slices of the subsequent station. Slice overlap, which is routinely applied e.g. in time-of-flight MRA or QISS, was not used; slices within a station were acquired with no overlap (distance factor = 0) to allow for a faster coverage. Separate from the parameters that were kept the same for all three techniques, the protocols were adapted individually to the shortest TE/TR possible without affecting image quality/contrast.

Currently we are limited to 8 kW power which is not enough to perform abdominal imaging with arbitrary flip angles. Here, we used 80% of the maximum available power or 100% of the available peak power (which in this case corresponds to a nominal flip angle of 80° in the protocol). We concluded from preceding volunteer measurements that this power level gives us satisfying MRA contrast.

#### GRE

TR was set to 14 ms, TE to 5.26 ms, and the bandwidth to 1300 Hz/px. Image slices were acquired sequentially and in ascending order (feet to head) additionally using flow compensation. An RF pulse of nominal 90° for the suppression of the veins was applied every TR with a thickness of 116 mm and positioned at a distance of 10 mm below the imaging slice, travelling with the current slice position. TIAMO imaging [15], [16] used CP^+^ and CP^2+^ except for the stations of the upper thigh in volunteers where dedicated TIAMO shims had to be calculated. Hence, every slice was acquired twice. The TIAMO trigger changes excitation mode after every excitation RF pulse. The total acquisition time per station (12 cm coverage) amounted to 2 min 41 s.

#### UGRE

A TR of 700 ms per slice, a TE of 3.45 ms, and a bandwidth of 930 Hz/px were chosen. Image slices were acquired sequentially and in ascending order using flow compensation. Due to shorter flow compensation gradients, a shorter TE than in GRE could be reached; UGRE also used shorter spoiling gradients for remaining transversal magnetization than GRE. No inversion RF pulses for contrast preparation were used. The same RF pulse settings for venous suppression as in the GRE protocol were used except that the duration was only 2000 µs instead of 3840 µs and a pulse was applied only every 2^nd^ excitation and using a shortened spoiler gradient of 1 ms length with a gradient moment of about 18 ms mT/m compared to 135 ms mT/m and ∼6 ms duration in the original implementation and 15 ms mT/m in ∼0.5 ms in GRE. TIAMO imaging [15], [16] again used the CP^+^ and CP^2+^ modes except for the stations of the upper thigh in volunteers where dedicated TIAMO shims had to be calculated. The sequence was gated by using the acoustic cardiac triggering device in such a way that one complete slice was acquired after the trigger signal. Due to TIAMO, each slice has to be acquired twice to combine both modes. In this protocol the two modes are acquired consecutively. After a single trigger signal, one complete slice is acquired in the first mode before the same slice excited by the second mode follows after the next trigger event. Total acquisition time for each station was on average ∼3 minutes, depending on the heart rate of the volunteer. Without cardiac triggering, 1 min 24 s would be achievable.

#### QISS

A TR of 800 ms per slice, a TE of 2.74 ms, and a bandwidth of 183 Hz/px was applied. Image slices were acquired sequentially with GRE readout using a partial Fourier factor of 5/8. After the saturation RF pulses, a subsequent time interval (QI, here, 350 ms) is applied to wait for non-saturated blood spins to enter the imaging region. The sequence was gated using the acoustic cardiac triggering device. A time delay of 100 ms followed the trigger event. As in UGRE, a single slice is completely acquired beginning with the trigger event. Total acquisition time for each station was on average ∼2 minutes depending on the heart rate of the volunteer. Without cardiac triggering, 48 s would be achievable. The protocol was run with only the CP^+^ mode applied to the 16-ch coil and only 1 average (instead of the 2 that were necessary for TIAMO in the other protocols). An overview of the relevant sequence parameters of GRE, UGRE and QISS can be found in Table 1.

**Table 1:** Sequence parameters.

	GRE	UGRE	QISS
**TR [ms]**	14	700 per slice	800 per slice
**TE [ms]**	5.26	3.84	2.74
**Bandwidth [Hz/px]**	1300	930	183
**Slice orientation**	Transversal	Transversal	Transversal
**Slices**	60	60	60
**Slice acquisition**	Sequential/ascending	Sequential/ascending/single shot	Sequential/ascending/single shot
**FOV [mm^2^]**	390×293	390×293	390×293
**Voxel volume [mm^3^]**	1.0×1.0×2.0	1.0×1.0×2.0	1.0×1.0×2.0
**Acquisition matrix**	384×288	384×288	384×288
**Parallel imaging with GRAPPA: acceleration factor/reference lines**	4/32	4/32	4/32
**Partial Fourier factor**	-	-	5/8
**Fat suppression**	-	-	Yes
**Venous saturation**	Prior to every excitation	Prior to every 2^nd^ excitation	Prior to single-shot slice acquisition
**Cardiac triggering**	-	Phonocardiogram	Phonocardiogram
**TIAMO**	Yes: CP^+^/CP^2+^ (dedicated shims in the upper thigh if necessary)	Yes: CP^+^/CP^2+^ (dedicated shims in the upper thigh if necessary)	No: CP^+^ (dedicated shim in the upper thigh if necessary)
**Averages (TIAMO)**	2	2	1
**TA per station [min∶s]**	2∶41	1∶24≤TA≤∼4∶00 2∶54 on average	0∶48≤TA≤∼3∶30 2∶09 on average

Overview of relevant protocol parameters for GRE, UGRE and QISS sequence. The total acquisition time TA of GRE and QISS depended on the individual's general heart rate and amounted to max about 4 min/3 min 30 s; without cardiac triggering 1 min 24 s/48 s would be necessary.

To compare the three sequences, 10 volunteers (4 female and 6 male, average age: 26.7 years, range: 20 to 38 years; average weight: 70.7 kg, range: 53 to 90 kg; average height: 1.75 m, range: 1.65 to 1.87 m) were examined by acquiring all three sequences consecutively at the same station before moving the AngioSURF table to the next station beginning at the hip and proceeding toward the feet.

For quantitative comparison of the three sequences, ROIs were placed in a single artery and in the tissue directly next to that artery for each station in both legs individually. The same artery and tissue positions were chosen for every sequence. Using the two ROIs, a vessel-to-background ratio (VBR = Signal_Vessel_/Signal_Tissue_ [31]) could be calculated, where values >1 are desirable to distinguish vessels, especially when looking at MIPs. The higher the VBR, the better the visibility of the vessel. For each station, the VBR was determined at 3 positions within the imaged region: In the central slice as well as in the slices located 5 slices toward the borders of the imaged region to avoid effects caused by gradient nonlinearity or B_1_ inhomogeneities which are expected to be especially apparent in the outermost slices. As in the QISS sequence TIAMO could not be used, to minimize the influence of B_1_ inhomogeneity in the evalutation of the QISS sequence, arteries were chosen that were not impaired by B_1_ inhomogeneities.

To check for statistical significant differences between the three sequences, a t-test was performed [32].

## Results

**Figure 2:**
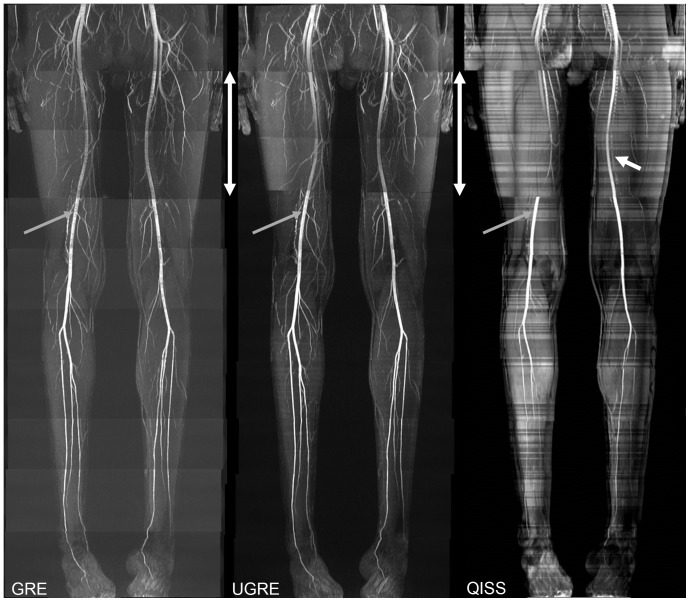
Complete MIPs of GRE, UGRE and QISS of one volunteer. MIPs of all acquired stations merged together manually in one volunteer for GRE, UGRE, and QISS. In this volunteer, the two stations covering the upper part of the thighs (marked by white double-headed arrows) used individual RF shims to prevent complete artery signal dropout. Vessel signal fluctuation in GRE could be mostly avoided in UGRE and QISS due to cardiac triggering (grey arrows). Tissue signal intensity fluctuation in QISS is most probably due to time delay induced by heartbeat triggering giving the surrounding tissue time to relax. White arrow in QISS shows vein visible in QISS sequence, as only one of the TIAMO RF shim modes could be used, preventing complete venous suppression.

In seven volunteers 10 stations were acquired, in two volunteers 11 stations, and in one volunteer 12 stations. On average 124 ROIs were placed per volunteer and sequence. In total, 1242 ROIs per sequence were evaluated. Figure 2 presents maximum intensity projections (MIPs) of all acquired stations for one volunteer. For the UGRE sequence, the sparse application of the venous saturation pulses combined with the shorter spoiler gradient and saturation RF pulse permitted acquisition of one complete slice across two heartbeats. Owing to TIAMO, two averages needed to be acquired to utilize both excitation modes. Due to the consecutive acquisition of both modes, excitation and saturation pulses are always run in the same mode. Nevertheless, sufficient suppression of the venous system could be realized in the final image. The inherent saturation effects of the GRE readout in the UGRE permitted suppression of the static background tissue including fat, even though no fat suppression RF pulses were applied.

The individual heart rate dictates the total acquisition time for triggered sequences, but on average, UGRE imaging took 2 min 54 s per station while QISS took only 2 min 9 s. Without triggering 36 s would be the difference between the two sequences (although UGRE acquires two acquisitions), amounting to 45 s on average when using cardiac triggering. This means, QISS was prolonged by a factor of 2.7 on average while UGRE is prolonged by a factor of 2.1 compared to imaging without any triggering.

As observed in [17], the use of a general B_0_ shim for all stations as determined at the uppermost station did not lead to perceivable image distortions.

For all volunteers, VBR was averaged over all stations and both legs using the standard deviation of the signal intensities inside the measured ROIs as error. For every volunteer UGRE performed better than GRE, while QISS achieved the lowest values in all volunteers (Table 2). In GRE and UGRE, fluctuation of VBR between different slices within a station as well as in between different stations is much higher than in QISS imaging. This is reflected in the overall higher errors for the calculated VBR in Table 2. Highest vessel signal could be observed in the QISS sequence, but also the highest tissue signal, which taken together led to the lowest VBR. Between different stations no relevant deviation and no tendency within a station (e.g. high VBR in uppermost slice and then decrease in signal) could be observed, except that vessels were harder to identify in the lowest stations at the level of the feet. In four volunteers for all sequences, delineation of arteries was not possible in the complete lowermost station, and in three volunteers delineation was not possible in the lowermost slices. In QISS images identification of the arteries in the lowermost station was not possible in four volunteers (in one of them also not possible for GRE). Averaged over all volunteers (and stations), GRE reached a VBR of 4.18±0.05, UGRE 5.20±0.06, and QISS 2.72±0.03 (Table 2).

**Table 2:** Averaged VBR of all volunteers.

	V1	V2	V3	V4	V5	V6	V7	V8	V9	V10	Average
**GRE**	4.53±0.17	4.22±0.17	4.85±0.26	3.69±0.12	3.08±0.16	4.47±0.17	4.17±0.17	3.46±0.14	5.42±0.17	3.85±0.13	**4.18±0.05**
**UGRE**	4.90±0.19	6.08±0.23	5.56±0.30	4.90±0.16	3.46±0.13	5.44±0.21	5.88±0.21	4.82±0.19	5.58±0.19	5.37±0.17	**5.20±0.06**
**QISS**	2.47±0.08	3.29±0.16	2.24±0.10	3.11±0.08	1.85±0.06	2.96±0.14	3.23±0.11	3.51±0.11	2.29±0.05	2.26±0.05	**2.72±0.03**

Averaged VBR over all stations and both legs for the ten volunteers. In the far right column, the average over all volunteers is given. Standard deviation of the signal intensities inside the measured ROIs were used as error. Error values in this table were calculated via rules for propagation of uncertainty. The QISS sequence led to the lowest VBR in all cases, whereas UGRE performed best. Overall, UGRE reached relevantly higher values than GRE.

To test for statistically significant differences between the three techniques, a t-test was performed. The resulting P values provided an estimation for the significance of differences, and P values less than 0.05 can be considered as statistically significant. The results of the t-test showed that the differences between all techniques are statistically significant. Paired comparisons led to P = 0.02/0.03 for GRE compared to UGRE/QISS and P<0.001 for UGRE compared to QISS.

**Figure 3:**
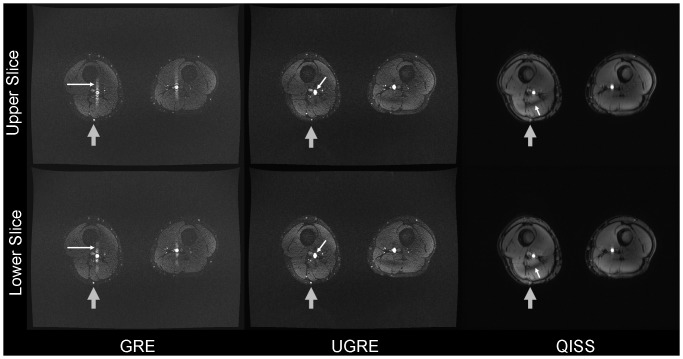
Signal variation flow artifact. Signal variation flow artifact (white arrow in GRE) shown for consecutive axial slices at position of the grey arrow in Fig. 1 compared to the corresponding images in UGRE and QISS. In UGRE large arteries seem to be slightly broadened (white arrow). In the QISS images, B_1_ inhomogeneities are considerably more pronounced as TIAMO could not be used (white arrows). For GRE and UGRE, especially the smaller vessels can be more easily delineated than for QISS (grey arrows). The upper row shows the slice position superior to the images of the lower row.

**Figure 4:**
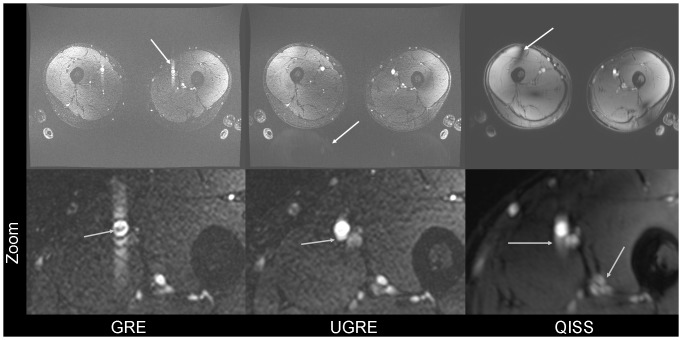
Artifacts in GRE, UGRE and QISS. The upper row visualizes the flow artifact in GRE images, aliasing in UGRE, and B_1_ inhomogeneity in QISS, in each case marked by a white arrow. Due to use of TIAMO, the B_1_ inhomogeneity could be ameliorated in GRE and UGRE. Zoomed images of the left thigh in the lower row show GRE, where the flow artifact in the phase-encode direction and a small signal dropout inside the vessel lumen are clearly visible (grey arrow), UGRE and QISS. For UGRE, slight broadening of the arteries can be observed (grey arrow). In the QISS image, blurring of the vessel walls is clearly observed (grey arrows). Artery circumference can be delineated best in UGRE.

Flow artifacts were very severe in GRE images (Fig. 3, 4), while in UGRE and QISS only a slight broadening in the phase-encode direction could be observed in some slices (Fig. 3). In UGRE images weak aliasing artifacts could be observed (Fig. 4). Since no TIAMO could be used for QISS imaging, clearly visible B_1_ inhomogeneities are present which are considerably less pronounced or not visible in GRE and UGRE (Fig. 3, 4). Additionally, vessel contours appeared slightly blurred in QISS imaging (Fig. 4). Also, veins are often visible whereas they are nearly completely suppressed in GRE and UGRE (Fig. 2). Due to the blurring and the lower VBR in QISS, vessels were more poorly delineated or weren't even visible (Fig. 3, 4).

**Figure 5:**
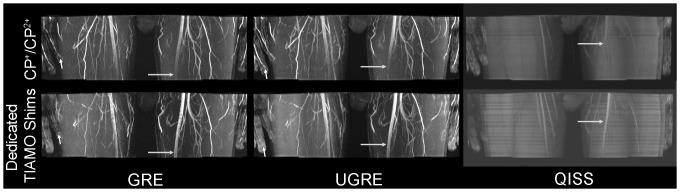
Dedicated TIAMO shims. MIPs showing the first station of the thigh. The upper row shows images acquired using TIAMO with the CP^+^ and CP^2+^ modes. In the case of QISS, only CP^+^ was used. In the lower row, dedicated TIAMO shims were calculated after the acquisition of B_1_ maps. In the case of QISS, only one of the two modes could be used. One can clearly see that the left superficial femoral artery can be delineated throughout the entire station with the dedicated B_1_ shims (grey arrows), whereas the finger arteries lost signal (short white arrow in GRE and UGRE).

In six volunteers (3 female, 3 male), dedicated TIAMO shims for the upper thighs were calculated based on individual B_1_ maps to improve the visibility of the arteries (Fig. 5). After detection of the impaired vessel visibility, the scan was interrupted and restarted with the new shims after B_1_ maps were acquired and the dedicated RF shims were calculated. The acquisition of the B_1_ maps and the calculation took less than 2 minutes. The remaining stations were examined with the CP^+^ and CP^2+^ modes. The use of TIAMO imaging in conjunction with dedicated shims in the upper thighs of certain volunteers led to nearly homogenous images over all stations.

## Discussion

The use of cardiac triggering in conjunction with both the UGRE and QISS sequences suppressed intravascular signal variations [17] at the cost of measurement time. The triggering itself (due to time intervals between two triggers) and variations in current heart rate prolong the measurement time depending on the individual's general heart rate. If the succeeding trigger event occurs too early and k-space data sets are not acquired completely, a time delay until the next trigger event must be introduced. The acquisition time per station ranged from approximately 1 min 30 s to 3 min. In the QISS sequence, the gating and the thereby induced time delay is reflected in signal variations of the background tissue (cf. Fig. 2). A very fast heart rate could also potentially lead to a relevant amount of interrupted data acquisition due to the gating. This would require triggering on only every 2^nd^ heartbeat, prolonging the measurement time even more. Especially for fast heart rates, the possibility to use the saturation pulses sparsely as well as the shorter spoiler gradient and the shorter saturation RF pulse were essential. For the given UGRE protocol, the fastest possible heart rate is 85 bpm, limited by the inverse of the TR of 700 ms. The TR also determines the acquirable image resolution and correspondingly holds for the QISS sequence which is reflected in the factor the total acquisition time per station was prolonged (2.1 for a TR of 700 ms in the UGRE and 2.7 for a TR of 800 ms in the QISS). To speed up the UGRE sequence further, a lower resolution or a more sparsely applied saturation sequence segment would be possible. However, the latter would sooner or later lead to more pronounced vein visibility. Further investigation of the relationship between heart rate and the necessary minimum number of applied saturation pulses could be pursued, but a relevant benefit is not expected. The pragmatic way to acquire data from volunteers with higher heart rates would be to trigger only every 2^nd^ heartbeat, concomitantly enabling a resolution increase at the cost of a longer acquisition time. A general increase of the image resolution would require sparser triggering, depending on the heart rate.

The use of a general B_0_ shim that was determined at the uppermost station was robust enough to be used for all following stations without perceivable image distortions, thus helping to moderate total acquisition time as the B_0_ shim procedure did not need to be repeated.

The comparison of the measured VBR shows that the UGRE sequence performed best, as it showed only moderate artifacts and led overall to the highest VBR of all sequences. It provided the best artery visibility of all sequences, as the GRE was impaired by flow artifacts combined with signal dropouts due to the lack of triggering. In one of the 10 volunteers GRE performed equally to UGRE in terms of VBR (V9 in Table 2), but UGRE was still preferable as GRE was strongly impaired by flow artifacts. The QISS protocol showed a comparatively smaller number of vessels with blurred contour. UGRE performed statistically significant better than GRE and QISS, while VBR for UGRE and GRE were quite comparable and clearly higher than for QISS. The fact that the vessel signals acquired with QISS were the highest of all sequences shows the potential of this sequence. Unfortunately, the preparation RF pulses do not seem to work as effective as for lower field strength in suppressing the background tissue. As the QISS protocol is a vendor-provided work-in-progress at 7T, the actual VBR can only be treated as preliminary result and could improve with further optimizations at this field strength; in particular, implementation of the TIAMO technique would be expected to significantly reduce B_1_ artifacts.

In the lowermost station at the level of the feet, depiction of the arterial vessels was not possible in all slices (in seven cases for all sequences). Here, a higher imaging resolution, at the cost of prolonged total acquisition time, might help to identify more vessels. Apart from the narrow vessel diameters near the feet, a technical reason for this problem could be a relevant difference in loading of the RF coil with the feet compared to the region of the pelvis.

The absence of heartbeat triggering in the GRE sequence led to the same flow artifacts as reported in [17], which are most probably induced by an interference between individual heart rate and image acquisition frequency. GRE images are acquired covering the whole cardiac cycle and therefore a strong variation in flow velocity is present which leads to differences in flow compensation efficiency. This issue can be avoided mostly in UGRE and QISS via cardiac triggering. The weak aliasing artifacts that were visible in the UGRE sequence did not disturb the image quality, especially when looking at MIPs. The reason for the aliasing artifact is that the saturation RF pulse is applied only every second TR which leads to two different effective TRs. This leads to a periodic signal variation: Every second line in k-space is acquired with slightly lower signal which results in an artifact in axial slices which appears similar to aliasing. A possibility to prevent that would be to excite with two different flip angles and/or using a different k-space ordering. But as the artifact is only slightly visible in the axial images and not visible in the MIPs, this was not implemented yet and might be topic of future study. The slight vessel broadening visible in the phase-encode direction in UGRE was not visible in the coronal MIPs. The reason for the latter artifact is most probably that, despite cardiac triggering, the blood flow velocities are not constant during acquisition of the whole slice and thus the pronounced flow artifact observed in GRE remains in small parts visible in UGRE. In general, as two consecutively excited modes contribute to one individual slice the UGRE sequence is more prone to volunteer movements than GRE. On the other side, the consecutive acquisition ensures application of excitation and saturation pulses in the same mode, simplifying sparse application of the saturation pulses and leading to sufficient venous suppression in the final image. In the QISS images blurring of the vessels was observed which led to poorer vessel delineation, and especially smaller vessels could not be delineated at all. Regarding artifacts, UGRE would be the most preferable sequence.

Six of the examined volunteers required calculation of individual TIAMO RF shim settings for the station at the level of the thighs to improve the visibility of the superficial femoral artery in both legs; this extra step required the acquisition of B_1_ maps. Another possible solution for this issue may be the use of high-permittivity pads to increase the transmit field homogeneity [33]. The remaining stations could be examined with the CP^+^ and CP^2+^ modes, indicating that the combination of the CP^+^ and CP^2+^ mode is relatively robust for TIAMO imaging. The most probable reason for the need of individual RF shimming is the variable anatomy at the transition from pelvis to the legs along the longitudinal axis. Also, this might depend on the leg circumference and/or the ratio between fat and muscle.

In conclusion, the UGRE sequence variant performed best for non-enhanced MRA of the lower extremity vessels, as the observed artifacts were only moderate and the highest average VBR was reached with a statistically significant difference to the compared sequences. The QISS sequence performed worst regarding VBR, while GRE showed the most severe flow artifacts despite reaching satisfactory VBR values. This study also revealed that the calculation of individual RF shim settings in a large proportion of volunteers as well as cardiac triggering, both of which prolong measurement time, are essential for the continuous depiction of the lower extremity arteries with non-enhanced MRA at 7T.
